# Poster Session I - A180 MULTI-OMIC PROFILING REVEALS CHANGES IN HEPATIC TRANSCRIPTOME, METABOLOME, AND MICROBIOME ASSOCIATED WITH IMPROVEMENT IN HISTOLOGY AT 12 MONTHS POST ROUX-EN-Y GASTRIC BYPASS

**DOI:** 10.1093/jcag/gwaf042.180

**Published:** 2026-02-13

**Authors:** Y Ghorbani, K J Schwenger, H Maughan, A Teterina, W Lou, E M Comelli, S E Fischer, T Jackson, A Okrainec, J P Allard

**Affiliations:** University Health Network, Toronto, ON, Canada; University Health Network, Toronto, ON, Canada; National Coalition of Independent Scholars, Toronto, ON, Canada; University of Toronto Dalla Lana School of Public Health, Toronto, ON, Canada; University of Toronto Dalla Lana School of Public Health, Toronto, ON, Canada; University of Toronto Department of Nutritional Sciences, Toronto, ON, Canada; University Health Network, Toronto, ON, Canada; University Health Network, Toronto, ON, Canada; University Health Network, Toronto, ON, Canada; University Health Network, Toronto, ON, Canada

## Abstract

**Background:**

Prevalence of obesity-related metabolic dysfunction-associated steatotic liver disease (MASLD) has been increasing and its severe form of steatohepatitis (MASH) is expected to become the leading cause of liver transplantation. Roux-en-Y gastric bypass (RYGB) is an effective surgery for weight loss and improving MASLD. However, its impact on hepatic transcriptome (HT), intestinal microbiome (IM) and serum/fecal metabolome and relationship with changes in histology is not fully understood.

**Aims:**

To investigate changes in HT, serum/fecal metabolome and IM between pre- and 12 months post-RYGB and their relation to improvement in liver histology.

**Methods:**

In this prospective study, data was collected pre and 12 months post-RYGB and included clinical measurements, HT, serum/fecal metabolomes, IM, liver histology and Nonalcoholic Fatty Liver Disease Activity Score (NAS).

**Results:**

In 38 patients, HT analysis revealed a distinct co-expression module enriched in fatty acid metabolism with genes involved in mitochondrial and peroxisomal β-oxidation and the tricarboxylic acid (TCA) cycle, including SUCLG2. More upregulation of β-oxidation genes correlated with less reduction in NAS, ballooning, and inflammation while an increase in TCA cycle gene expression was linked to resolution of ballooning. Related to these changes, there was a significant increase in fecal acylcarnitines, likely due to malabsorption from RYGB, with a significant reduction in circulating acylcarnitines which correlated positively with SUCLG2 expression and improvement in ballooning. Also, the increase in fecal acylcarnitines positively correlated with the bacterial species capable of utilizing acylcarnitines. Network analysis showed that ballooning of hepatocytes was associated with several acylcarnitines and TCA metabolites while SUCLG2 was the hub gene associated with these changes.

**Conclusions:**

RYGB improves MASLD with significant changes in HT, serum/fecal metabolome and IM. These changes are mostly related to fatty acid metabolism, TCA cycle and acylcarnitine metabolism with ballooning being a central link between hepatic lipid oxidation pathways, related intermediates, and IM. Circulating acylcarnitines could potentially be a biomarker for MASH.

**Funding Agencies:**

CIHRAmerican College of Gastroenterology Bridge Award, BBDC Tamarack Graduate Award in Diabetes Research

